# Unequal benefits: housing provident fund and happiness among China's migrant and non-migrant populations

**DOI:** 10.3389/fpsyg.2026.1741178

**Published:** 2026-02-25

**Authors:** Yutong Wu, Weijun Li, Mingzhi Hu

**Affiliations:** 1 School of Finance, Zhongnan University of Economics and Law, Wuhan, China; 2 School of Business, Anhui University of Technology, Ma'anshan, China; 3 Chinese Academy of Housing and Real Estate, School of Management, Zhejiang University of Technology, Hangzhou, China

**Keywords:** China, equality, happiness, housing provident fund, migrant

## Abstract

This study examines whether China's Housing Provident Fund (HPF) improves subjective wellbeing equitably across urban populations. Using data from the China Household Finance Survey (CHFS), we ask two questions: (1) Does HPF participation enhance individuals' happiness? (2) Do its effects differ between migrant and non-migrant residents? Employing multivariate regression models with rich socioeconomic controls, we find that HPF participation is positively associated with overall happiness. However, this association is uneven: a statistically significant positive relationship is observed for non-migrants, while no statistically meaningful association is found for migrants. Mechanism analyses indicate that this disparity stems from migrants' limited ability to utilize HPF benefits, particularly housing loans, due to unstable employment and shorter job tenures that hinder eligibility and savings accumulation. These findings reveal that a policy designed as a universal housing support system produces unequal welfare outcomes. By shifting attention from average effects to distributional consequences, this study advances the literature on housing policy and subjective wellbeing and demonstrates how universal programs can reinforce existing social stratification in rapidly urbanizing societies.

## 1 Introduction

The pursuit of happiness is a fundamental human endeavor, deeply intertwined with various aspects of life, including one's living conditions (Hu et al., 2020; Oleńczuk-Paszel and Sompolska-Rzechuła, 2025). Homeownership, in particular, has been recognized as a significant contributor to subjective wellbeing by providing individuals with a sense of security, stability, and accomplishment (Hu and Su, 2026; Hu and Ye, 2020; Will and Renz, 2025). This connection between housing and happiness underscores the importance of examining policies and initiatives aimed at facilitating access to adequate housing.

The Housing Provident Fund (HPF) is a mandatory savings program designed to help employees save for housing-related expenses (Wu et al., 2025). Both employees and their employers contribute a certain percentage of the employee's salary to the fund periodically. By pooling resources, the HPF aims to improve housing affordability. Previous studies have highlighted the positive role of the HPF in enhancing happiness among urban residents (Chen et al., 2022). However, there is a notable gap in the literature regarding the equity aspects of this housing affordability policy. Existing research suggests that the HPF primarily benefits middle- and high-income contributors while excluding low-income individuals who lack sufficient purchasing power to take advantage of these preferential policies (Yu and Cui, 2021). Moreover, the structural imbalance in HPF participation often leaves temporary workers and those in flexible employment outside the HPF program (Chen et al., 2025).

This study aims to investigate the fairness of the HPF program by examining its differential impact on the subjective wellbeing of migrant and non-migrant populations in China. We focus on the Chinese context for several reasons. First, the HPF is a cornerstone of China's policy-oriented housing finance, with its scale continuously expanding since its pilot implementation in Shanghai in 1991 (Deng et al., 2021). It has become a crucial tool for urban workers to address housing challenges, serving as the primary means to improve housing conditions and enhance the wellbeing of low and middle-income groups (Chen et al., 2022). Through a mutual contribution mechanism that involves both employees and their employers making regular contributions to a dedicated fund, the HPF partially shifts the housing burden from employees, optimizing their financial allocation and supporting the realization of the “Chinese Dream” of adequate housing for all. Second, China has a substantial migrant population, characterized by individuals who move from rural areas to urban centers in search of better employment opportunities and living conditions. According to the National Bureau of Statistics of China, the country had approximately 292.51 million migrant workers in 2021, accounting for about 20.7% of China's total population of around 1.41 billion people.

Utilizing data from the China Household Finance Survey (CHFS), we find that the HPF significantly enhances residents' happiness, consistent with previous studies (Chen et al., 2022). However, our analysis reveals that HPF participation is positively associated with wellbeing among non-migrant populations, whereas no statistically significant association is observed for migrants. The mechanism analysis suggests that this disparity may be related to differences in HPF utilization. Eligibility for HPF loans typically requires continuous contributions for at least 6 months and stable employment (Cui et al., 2023; Zhao and Bourassa, 2003). For migrants, the absence of a significant association between HPF and happiness is plausibly linked to their lower likelihood of meeting these eligibility requirements and accumulating sufficient savings, given their relatively unstable employment conditions and shorter job tenures.

This study contributes to the existing literature on housing policies and subjective wellbeing in two key ways. First, it shifts the focus from overall outcomes to equity considerations in evaluating the HPF, addressing a critical gap in understanding the distributional effects of housing policies. Basically, the HPF enhances overall happiness by facilitating homeownership, reducing financial barriers, providing financial security, increasing disposable income, and supporting wealth accumulation through housing assets (Manturuk et al., 2017; Tang and Coulson, 2017; Wang et al., 2018; Xu, 2017). However, less attention has been paid to the equity effect of the housing affordability policy. Second, it adds to the broader discourse on urban social stratification by showing how universal housing policies can inadvertently reinforce existing socioeconomic inequalities. By demonstrating that the benefits of the HPF are not evenly distributed across urban populations, the research highlights the complex interplay between housing policies and subjective wellbeing.

The remainder of this paper is structured as follows: Section 2 provides a review of theories of happiness and develops the hypotheses. Section 3 outlines the data sources, variable construction, and empirical strategies employed in our analysis. Section 4 presents and discusses the empirical results, followed by a series of robustness checks and additional mechanism analyses. Finally, Section 5 concludes the study.

## 2 Theories and hypothesis development

### 2.1 Theories

Happiness emanates from an individual's psychological state, encompassing both emotional experiences and rational cognitive judgments. Happiness can be broadly categorized into two main types: hedonic wellbeing and eudaimonic wellbeing. Hedonic wellbeing leans toward emotional experience, viewing happiness as a collection of positive emotions formed by subjective and intrinsic feelings of satisfaction and identification (Atkinson, 2013). Its measurement indicators typically include degree of life idealization, living conditions, life satisfaction, sense of life achievement, and life identification (Zhang et al., 2024). In contrast, eudaimonic wellbeing represents an individual's cognitive judgment of their own value (Grant et al., 2007). In this dimension, happiness is understood as the self-discovery and self-recognition of personal development, potential, achievements, and existential meaning. Its measurement indicators includes two levels with six dimensions: personal growth, life meaning, autonomy, and self-worth at the individual level; positive interpersonal relationships and environmental mastery at the social level (Ryff and Singer, 2008).

This paper focuses on residents' subjective wellbeing, which originates from hedonic wellbeing (Angner, 2010). Subjective wellbeing refers to an individual's subjective experience of their current life state and quality based on certain life standards, reflecting people's overall assessment of their life satisfaction and happiness level. The current literature has extensively examined the factors influencing subjective wellbeing, which can be divided into individual and social perspectives. From an individual perspective, micro-factors influencing subjective wellbeing include basic personal characteristics, income level, and housing conditions. Personal characteristics encompass dimensions such as age, gender, health status, and education level (Blanchflower, 2021; Hu and Ye, 2020; Zweig, 2015). Income level can be further divided into absolute and relative income levels. Both absolute and relative income exhibit a positive and significant correlation with happiness, with changes in relative income exerting a greater impact on happiness than changes in absolute income (Brady et al., 2023). Regarding housing conditions, factors such as housing affordability, homeownership, and neighborhood environment significantly affect happiness (Chen et al., 2022; Hu and Ye, 2020; Somarriba Arechavala et al., 2022).

From a social perspective, factors such as residents' social status, employment stability, and income inequality significantly impact residents' happiness (Anderson et al., 2012; Knight and Gunatilaka, 2022; Yagi et al., 2016). The relationship between income inequality and individual happiness is proposed to be inverted-U shaped (Gao et al., 2022). This tunnel effect of income inequality on individual happiness is supported by Wang et al. (2015) using data from a national household survey in China. Additionally, indicators related to social security, such as social pensions, insurance, and welfare, have significant effects on residents' happiness (Fang and Sakellariou, 2016; Pak, 2020). Sustainable urban development also play crucial roles in affecting the happiness of residents (Cloutier et al., 2014).

### 2.2 Housing provident fund and happiness

The HPF program possesses long-term and mutual aid characteristics. Participation in the HPF should have a positive association with overall happiness through its dual role in facilitating homeownership and enhancing financial security. The HPF serves as a crucial mechanism that enables families to achieve homeownership more readily by providing accessible housing financing options (Tang and Coulson, 2017). For many households, particularly those from middle-income backgrounds or new urban families without prior housing, the HPF reduces financial barriers associated with purchasing a home (Wang et al., 2018). This accessibility not only improves their housing conditions but also fosters a sense of stability and belonging within their communities (Rohe and Stewart, 1996).

Furthermore, the HPF contributes significantly to residents' financial security. By offering low-cost housing loans and acting as a form of additional income through withdrawal options (Xu, 2017), the fund enhances disposable income and reduces financial uncertainty among participants. This stability translates into reduced stress related to housing expenses and a greater capacity to save and invest in other aspects of life, such as consumption, education and healthcare (Chen et al., 2020; Tang and Coulson, 2017). As residents feel more financially secure and empowered by their homeownership status facilitated by the HPF, their overall happiness levels tend to increase (Zhang et al., 2019; Zheng et al., 2020).

Moreover, the HPF system supports wealth accumulation through housing assets (Turner and Luea, 2009; Wainer and Zabel, 2020), which plays a pivotal role in enhancing economic stability and security for participant families. Owning a home not only provides a tangible asset but also shields households from fluctuations in rental costs and housing market dynamics. This long-term stability contributes to a sense of achievement and pride in homeownership (Manturuk et al., 2017), reinforcing positive emotions and overall wellbeing.

The HPF in China serves as a critical policy tool aimed at enhancing housing affordability and urban residents‘ overall wellbeing. However, its effectiveness varies notably between migrant and non-migrant populations, primarily due to disparities in their utilization of the HPF. To qualify for HPF loans, applicants typically must exhibit sustained commitment by contributing to the fund for at least 6 months while maintaining stable employment (Cui et al., 2023; Zhao and Bourassa, 2003). However, migrants encounter distinct obstacles in accessing and utilizing HPF benefits due to their frequently precarious employment situations and shorter job durations. As summaired by Swider (2015), migrants in individualized employment navigate high marginalization and hyper-mobility, embodying urban challenges posed by rising migration and informalized labor markets. These challenges hinder their ability to meet the criteria for HPF loans or accumulate sufficient savings through the fund, consequently restricting their capacity to afford housing in urban areas. Therefore, the potential positive impact of the HPF on migrants' wellbeing may be considerably subdued compared to their non-migrant counterparts, who can more readily leverage the fund's resources to secure stable housing and enhance their quality of life.

**Hypothesis 1** *Participation in the HPF is positively associated with overall happiness*.

**Hypothesis 2** *The positive effect of the HPF on happiness is less significant for migrants than for non-migrants*.

## 3 Data and methodologies

The empirical analysis in this study utilizes data from the China Household Finance Survey (CHFS), a comprehensive initiative conducted by the Survey and Research Center for China Household Finance at the Southwestern University of Finance and Economics. The CHFS employs a stratified, three-stage, probability proportional to size (PPS) sampling method to ensure representation of households across China. Data collection is facilitated through a Computer-Assisted Personal Interviewing (CAPI) system, capturing a wide array of information including demographics, socio-economic indicators, subjective wellbeing, HPF participation, and household assets and income. The CHFS has been conducted in five waves: 2011, 2013, 2015, 2017, and 2019. The initial round in 2011 encompassed approximately 8,438 households from 80 counties across 25 provinces. Subsequent rounds saw expansion, with the 2013 survey covering about 28,141 households. The scope further increased in 2015, sampling around 37,289 households. The 2017 round included about 40,011 households, and the most recent 2019 survey covered approximately 31,364 households.

Since only workers could participate in the HPF scheme, we restricted our analysis to working adults aged between 16 and 65. We further focused on urban areas because they typically present unique housing market dynamics and challenges that directly impact migrants' access to housing benefits. After excluding observations with missing values, the final dataset comprises 38,518 observations.

### 3.1 Happiness

In this study, the dependent variable is happiness. According to the response options for the question “Overall, do you feel happy now?” in the survey questionnaire, this study assigned values from 1 to 5 to the five options: “very unhappy,” “unhappy,” “average,” “happy,” and “very happy,” respectively. Statistical analysis of the data revealed that 48.50% of respondents reported feeling “happy,” and 15.89% indicated they were “very happy.” Additionally, 31.38% of participants described their happiness level as “average.” Conversely, 3.60% of residents considered themselves “unhappy,” and 0.63% reported feeling “very unhappy.” On average, the subjective happiness score for all respondents was 3.75, positioning it between “average” and “happy” on the scale.

### 3.2 HPF

The independent variable in this study is a binary indicator representing respondents' participation in the HPF program. This variable was constructed based on the question in the survey that asks, “Do you currently have a HPF account?” Respondents who answered “Yes” were assigned a value of 1, indicating their participation in the HPF scheme, while those who responded “No” were assigned a value of 0, indicating non-participation.

### 3.3 Migrant

To examine the heterogeneous effects of the HPF on the subjective wellbeing of different residential groups, this study divides the sample into migrant and non-migrant populations. Migrants are defined as residents whose current place of residence differs from their registered household location or who have been living outside their hometown for a period exceeding 6 months. This classification is operationalized through two specific questions in the survey: “Have you lived or worked in a location other than your registered residence for more than 6 months?” and “Is your household registration in the town/street where you currently reside?” If a respondent answers “yes” to the former or “no” to the latter, they are categorized as a migrant.

We control for various individual and household characteristics consistent with previous research to isolate the impact of the HPF on happiness. Our model incorporates age and its squared term, as age influences life satisfaction and priorities at different stages of life. Gender is included as a binary variable (female = 1, male = 0) to account for potential differences in happiness stemming from societal roles and expectations. Marital status (married = 1, otherwise = 0) is another essential control variable, as married individuals often report higher levels of happiness due to companionship and support. Education (college and above = 1, otherwise = 0) influences an individual's cognitive and social resources, affecting overall life satisfaction. Political status (Communist = 1, otherwise = 0) reflects ideological alignment with governing principles, potentially impacting perceived social equity and benefits. Health status (good health = 1, otherwise = 0) is a direct predictor of happiness, as good health enhances quality of life. Household size, measured by the number of family members, can influence happiness through social support and economic burden. Household income, measured in yuan over the past year, directly impacts living standards and financial security. Lastly, household wealth, represented by the value of household assets, provides a buffer against economic shocks and contributes to long-term security and happiness. Table 1 reports the summary statistics of these variables in the full sample as well as in the subsamples of migrants and non-migrants. Unconditionally, the happiness score of households with HPF is higher than that of households without HPF (3.810 vs. 3.710), and the difference is statistically significant at the 1% level. Additionally, significant differences exist in all household characteristics between these two groups.

**Table 1:** Summary statistics.

**Variables**	**Full sample**	**Households with HPF**	**Households without HPF**	**Mean difference**	***p*-value**
Happiness	3.754	3.805	3.710	0.095	0.000
HPF	0.466				
Age	41.885	41.362	42.342	−0.980	0.000
Female	0.417	0.385	0.445	−0.060	0.000
Married	0.909	0.917	0.902	0.015	0.000
College	0.256	0.440	0.095	0.345	0.000
Communist	0.231	0.361	0.118	0.243	0.000
Health	0.620	0.653	0.590	0.063	0.000
Household size	2.827	2.688	2.948	−0.260	0.000
Household income	108,824	138,971	82,538	56,433	0.000
Household wealth	1,357,198	1,737,925	1,025,243	712,682	0.000

*N* = 38518. Data source: China Household Finance Survey 2011, 2013, 2015, 2017, and 2019.

### 3.4 Empirical strategy

To test Hypothesis 1, which suggests a positive effect of HPF on happiness, we estimate the regression model with the following form:


$$\begin{array}{l} Happiness=\beta_{0}+\beta_{1}HPF+Controls+\vartheta_{region}+\sigma_{year}+\varepsilon \end{array}$$ (1)

where the dependent variable Happiness denotes respondents' reported levels of happiness ranging from very unhappy (1) to very happy (5). The independent variable HPF is an indicator variable that equals 1 if the respondent participates in the HPF. Controls denote a vector of control variables detailed in Table 1. ϑ_*region*_ and σ_*year*_ represent province and year fixed effects, respectively. Finally, ε is the error term. The standard errors are clustered at the province level to account for potential within-province correlations and heteroskedasticity. We used the linear probability model to estimate Equation (1) as it simplifies the estimation process and interpretation and generates similar results with non-linear models. As robustness checks, we also used the ordered logit and probit models, and the results remain consistent.

To examine Hypothesis 2, which posits a differential effect of the HPF on happiness between migrant and non-migrant populations, we re-estimate Equation (1) using two distinct subsamples: migrants and non-migrants. Furthermore, we investigate potential reasons for the varying impacts of the HPF, considering the disparities in the utilization of HPF among migrant and non-migrant populations. The model takes the following form:


$$\begin{array}{r} Limited\;HPF\;utilization=\\ \beta_{0}+\beta_{1}Migrant+Controls+\vartheta_{region}+\sigma_{year}+\varepsilon \end{array}$$ (2)

where Limited HPF utilization is proxied by three variables: instable occupation, Short employment duration, and Homeownership. The CHFS survey includes a question about respondents' employment, categorizing it into four types: permanent employees, long-term contract workers, short-term or temporary contract workers, and those without contracts. Based on responses to this question, we construct a binary variable Instable occupation. This variable is assigned a value of 1 for respondents who report being short-term or temporary contract workers or without contracts, and 0 otherwise. Additionally, respondents in the survey are asked about the duration of their current employment. Considering that one can only apply for an HPF loan after continuously contributing to the HPF for 6 months or more in most cities, we create another indicator variable Short employment duration, which is coded as 1 if the reported employment duration is less than 6 months, and 0 otherwise. Homeownership is an indicator variable for respondents owning a home in local cities. Migrant is an indicator variable for migrants. Other variables are defined the same as those in Equation (1).

## 4 Empirical results

### 4.1 Baseline results

Table 2 presents the regression results from Equation (1). To verify the consistency of estimators, this study progressively adds control variables and fixed effects to the regression model. Results in Column (1) show that without any control variables or fixed effects, the coefficient of HPF is positive and significant at the 1% level. This indicates that having access to the HPF significantly enhances residents' subjective wellbeing. Column (2) incorporates control variables for household and regional characteristics, and Column (3) further includes province and year fixed effects. The results in Columns (2) and (3) show that the coefficient of HPF remains positive and significant. Overall, after controlling for observables, the HPF is associated with a 0.028-point increase in the happiness scale, which is statistically significant at the 1% level. This finding supports Hypothesis 1.

**Table 2:** Housing provident fund and happiness.

**Variables**	**(1)**	**(2)**	**(3)**
	**Full sample**	**Full sample**	**Full sample**
HPF	0.095^***^	0.029^**^	0.028^***^
	(0.012)	(0.011)	(0.009)
Age		−0.043^***^	−0.045^***^
		(0.006)	(0.005)
Age-squared		0.0005^***^	0.0005^***^
		(0.0001)	(0.0001)
Female		0.014^**^	0.014^**^
		(0.005)	(0.005)
Married		0.292^***^	0.276^***^
		(0.026)	(0.023)
College		−0.068^***^	−0.065^***^
		(0.018)	(0.018)
Communist		0.088^***^	0.085^***^
		(0.009)	(0.008)
Health		0.313^***^	0.315^***^
		(0.009)	(0.009)
Household size		0.001	0.013^**^
		(0.005)	(0.005)
Household income		0.009^**^	0.007^*^
		(0.004)	(0.004)
Household wealth		0.059^***^	0.066^***^
		(0.006)	(0.005)
Region fixed effect	NO	NO	YES
Year fixed effect	NO	NO	YES
Observations	38,518	38,518	38,518
R-squared	0.004	0.073	0.088

Standard errors are reported in parentheses.

^*^ indicate significance at the 10% level.

^**^ indicate significance at the 5% level.

^***^ indicate significance at the 1% level.

**Figure 1:**
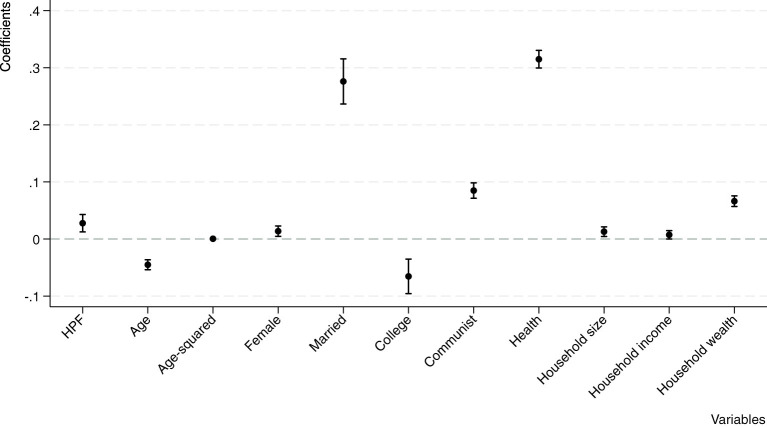
Estimated associations between HPF, demographics, and happiness. The vertical bars that extend beyond the markers represent the 10% confidence interval.

The results of the control variables align with expectations. The relationship between age and happiness exhibits a “U-shape” pattern (Blanchflower, 2021; Zhang et al., 2019). This implies that residents' happiness initially decreases with age but subsequently increases, displaying distinct life-cycle characteristics consistent with existing literature. Female respondents generally report higher levels of subjective wellbeing compared to males (Zhang et al., 2019; Zweig, 2015), possibly due to greater family and social responsibilities shouldered by men, which may lead to increased anxiety. Marriage is positively associated with happiness because it provides emotional support, companionship, and a sense of stability (Hu et al., 2020; Zheng et al., 2020). Highly educated people in China are less happy due to greater work stress, high expectations, and societal pressures (Wu et al., 2022). Communist Party members experience higher levels of happiness due to their access to privileges and social benefits, which significantly enhance their overall quality of life (Wang et al., 2015; Wu et al., 2022). Additionally, factors such as household income, household wealth, and personal health conditions all show positive correlations with residents' happiness. Figure 1 shows the estimated associations between HPF, demographics, and happiness.

Given that the primary beneficiaries of the HPF are local residents, this study further examines the differential impact of the HPF on non-migrant and migrant populations. Compared to non-migrants, migrants often find themselves “in the city but not of the city,” since they do not enjoy equal civic rights. This limitation restricts the ability of the HPF to improve their quality of life. For instance, migrants face constraints related to household registration and social insurance contribution duration when purchasing urban housing, thereby increasing barriers to utilizing the HPF for home acquisition and potentially suppressing improvements in their subjective wellbeing.

We divide the sample into migrant and non-migrant populations, with subsample regression results based on Equation (1) reported in Table 3. The findings indicate that within the non-migrant sample, the coefficient of HPF is positive and statistically significant at the 5% level. Conversely, in the migrant sample, the coefficient of HPF is not statistically significant. These results suggest a substantial disparity in the effect of the HPF on residents' happiness. Specifically, the HPF significantly enhances the subjective wellbeing of non-migrants while having no impact on migrants. This finding lends support to Hypothesis 2.

**Table 3:** Housing provident fund and happiness among migrants and non-migrants.

**Variables**	**(1)**	**(2)**	**(3)**
	**Full sample**	**Migrants**	**Non-migrants**
HPF	0.028^***^	0.032	0.025^**^
	(0.009)	(0.023)	(0.010)
Age	−0.045^***^	−0.052^***^	−0.044^***^
	(0.005)	(0.008)	(0.006)
Age-squared	0.0005^***^	0.001^***^	0.0005^***^
	(0.0001)	(0.0001)	(0.0001)
Female	0.014^**^	0.037	0.004
	(0.005)	(0.022)	(0.005)
Married	0.276^***^	0.299^***^	0.269^***^
	(0.023)	(0.035)	(0.026)
College	−0.065^***^	−0.075^**^	−0.063^***^
	(0.018)	(0.029)	(0.018)
Communist	0.085^***^	0.094^***^	0.082^***^
	(0.008)	(0.023)	(0.010)
Health	0.315^***^	0.323^***^	0.312^***^
	(0.009)	(0.018)	(0.011)
Household size	0.013^**^	0.014	0.011^*^
	(0.005)	(0.009)	(0.006)
Household income	0.007^*^	−0.005	0.012^***^
	(0.004)	(0.007)	(0.004)
Household wealth	0.066^***^	0.070^***^	0.066^***^
	(0.005)	(0.010)	(0.006)
Region fixed effect	YES	YES	YES
Year fixed effect	YES	YES	YES
Observations	38,518	8,244	30,274
R-squared	0.088	0.096	0.087

Standard errors are reported in parentheses.

^*^ indicate significance at the 10%, level.

^**^ indicate significance at the 5% level.

^***^ indicate significance at the 1% level.

Results from Table 3 are estimated using linear probability models. While linear probability models provide a straightforward interpretation of the relationship between independent variables and the probability of an outcome, they have inherent limitations that may lead to potential biases from model misspecification. One major limitation of linear probability models is that they assume a linear relationship between the independent variables and the probability of the outcome, which can result in predicted probabilities that are outside the [0,1] interval.

To address these concerns, we used ordered probit and logit models to re-estimate Equation (1) in both the full sample and subsamples of migrants and non-migrants. Ordered probit and logit models take into account the ordinal nature of the dependent variable by modeling the cumulative probabilities of the outcomes and ensuring that the predicted probabilities lie within the [0,1] range. While both ordered logit and ordered probit models are designed for ordinal dependent variables, they differ in their underlying distributional assumptions. The logit model assumes a logistic distribution of the error terms, whereas the probit model assumes a normal distribution. The results estimated by the ordered probit and logit models, as reported in Table 4, remain consistent. Overall, HPF participation is positively associated with happiness in the full sample, while this association is not statistically significant for migrants.

**Table 4:** Estimated results from ordered probit/logit models.

**Ordered probit models**	**(1)**	**(2)**	**(3)**
	**Full sample**	**Migrants**	**Non-migrants**
HPF	0.038^***^	0.044	0.034^**^
	(0.013)	(0.033)	(0.015)
Controls	YES	YES	YES
Region fixed effect	YES	YES	YES
Year fixed effect	YES	YES	YES
Observations	38,518	8,244	30,274
R-squared	0.039	0.043	0.039
**Ordered logit models**	**(4)**	**(5)**	**(6)**
	**Full sample**	**Migrants**	**Non-migrants**
HPF	0.063^**^	0.072	0.056^**^
	(0.025)	(0.059)	(0.028)
Controls	YES	YES	YES
Region fixed effect	YES	YES	YES
Year fixed effect	YES	YES	YES
Observations	38,518	8,244	30,274
R-squared	0.040	0.043	0.040

Standard errors are reported in parentheses.

^**^ indicate significance at the 5% level.

^***^ indicate significance at the 1% level.

### 4.2 Potential issues

#### 4.2.1 Omitted variable bias

Despite controlling for many household characteristics and regional and year fixed effects in the baseline models, concerns about omitted variable bias persist. This is because some unobservable factors that influence individual happiness may not be included in the models. We control for province-year fixed effects to mitigate omitted variable bias by accounting for time-varying unobservables at the province level. Specifically, province-year fixed effects help to control for all unobservable factors that vary across provinces over time but remain constant within a province in a given year. This includes economic conditions, policy changes, cultural shifts, and environmental factors that could influence happiness.

Table 5 reports the results by including province-year fixed effects from Equation (1) using the full sample, the subsample of migrants, and the subsample of non-migrants. The results in Column (1) show that, after controlling for time-varying regional unobservables, HPF participation is positively and significantly associated with happiness. Columns (2) and (3) further indicate that this association is not statistically significant for migrants, while it remains significant for non-migrants.

**Table 5:** Estimated results controlling for time-varying regional unobservables.

**Variables**	**(1)**	**(2)**	**(3)**
	**Full sample**	**Migrants**	**Non-migrants**
HPF	0.025^***^	0.028	0.023^**^
	(0.009)	(0.022)	(0.010)
Controls	YES	YES	YES
Region fixed effect	YES	YES	YES
Year fixed effect	YES	YES	YES
Observations	38518	8244	30274
R-squared	0.092	0.110	0.091

Standard errors are reported in parentheses.

^**^ indicate significance at the 10% level.

^***^ indicate significance at the 1% level.

#### 4.2.3 Sample selection bias

Since only workers could participate in the HPF scheme, we previously restricted our analysis to workers. However, this restriction may introduce sample selection bias as the analysis excludes non-workers who might have systematically different characteristics and happiness levels compared to workers. In other words, sample selection bias arises when the sample used in the analysis is not representative of the broader population, leading to biased and inconsistent estimates. In this context, restricting the sample to workers participating in the HPF scheme overlooks the experiences of non-workers who might be subject to different economic conditions, social environments, and personal circumstances, all of which can impact happiness. This exclusion potentially distorts the estimated relationship between HPF participation and individual happiness.

To address this bias, we used the Heckman selection model to correct for sample selection bias by considering the possibility of participating in the HPF scheme (i.e., being a worker) as a two-step process. The first step models the probability of being a worker (selection equation), while the second step models the happiness outcome conditional on being a worker (outcome equation). By incorporating both workers and non-workers in the analysis, the Heckman selection model corrects for the non-random selection into the worker sample, thereby providing unbiased and consistent estimates of the effect of the HPF scheme on happiness. Again, the results estimated by the Heckman selection models, as reported in Table 6, suggest that the effect of the HPF is non-significant for migrants.

**Table 6:** Estimated results from Heckman selection models.

**Variables**	**(1)**	**(2)**	**(3)**
	**Full sample**	**Migrants**	**Non-migrants**
HPF	0.029^***^	0.031	0.026^***^
	(0.008)	(0.023)	(0.010)
IMR	0.032	−0.023	0.022
	(0.021)	(0.065)	(0.024)
Controls	YES	YES	YES
Region fixed effect	YES	YES	YES
Year fixed effect	YES	YES	YES
Observations	50671	9740	40931

Standard errors are reported in parentheses.

^**^ indicate significance at the 5% level.

^***^ indicate significance at the 1% level.

### 4.3 Mechanism analysis

We have observed an insignificant impact of the HPF on migrants‘ happiness. This section investigates one potential explanation for this finding, focusing on disparities in HPF utilization between migrant and non-migrant populations. Eligibility for HPF loans typically requires consistent fund contributions over 6 months and stable employment (Cui et al., 2023; Zhao and Bourassa, 2003). We posit that the insignificant HPF effect on migrants' happiness stems from their limited access to HPF benefits.

To explore this hypothesis, we estimated Equation 2, with results reported in Columns (1) and (3) of Table 7. Our analysis supports this hypothesis, indicating that migrants are generally more likely than non-migrants to have unstable employment. Additionally, migrants tend to have shorter job tenures, further restricting their access to HPF benefits. Column (3) provides direct evidence that migrants face substantial barriers to homeownership in host cities, possibly due to disproportionate housing costs relative to their income levels. Columns (4)–(6) demonstrate that unstable employment and shorter job durations are negatively associated with happiness, whereas homeownership is positively correlated with happiness. These findings collectively suggest that limited HPF utilization among migrants is a significant factor contributing to the observed insignificant impact of the HPF on their happiness.

**Table 7:** Mechanism analysis.

**Variables**	**(1)**	**(2)**	**(3)**
	**Unstable occupation**	**Short employment duration**	**Homeownership**
HPF	0.090^***^	0.020^***^	−0.246^***^
	(0.010)	(0.004)	(0.026)
Controls	YES	YES	YES
Region fixed effect	YES	YES	YES
Year fixed effect	YES	YES	YES
Observations	38518	38518	38518
R-squared	0.106	0.046	0.188
	**(4)**	**(5)**	**(6)**
	**Happiness**	**Happiness**	**Happiness**
Unstable occupation	−0.034^**^		
	(0.014)		
Short employment duration		−0.070^**^	
		(0.026)	
Homeownership			0.100^***^
			(0.016)
Controls	YES	YES	YES
Region fixed effect	YES	YES	YES
Year fixed effect	YES	YES	YES
Observations	38518	38518	38518
R-squared	0.078	0.079	0.080

Standard errors are reported in parentheses.

^**^ indicate significance at the 5% level.

^***^ indicate significance at the 1% level.

## 5 Conclusion

This study provides a comprehensive examination of the differential impacts of HPF participation on the subjective wellbeing of migrant and non-migrant populations in urban areas. Our analysis demonstrates that while the HPF generally enhances residents' happiness, consistent with previous studies, this positive effect is primarily observed among non-migrant populations. In contrast, migrant populations show no significant improvement in subjective wellbeing from HPF participation. This disparity underscores the uneven distribution of benefits from what is intended to be a universal housing support system. Further analysis shows that the insignificance of the HPF on happiness for migrants is due to obstacles in utilizing the HPF, stemming from their precarious employment situations and shorter job durations, which hinder their ability to fulfill loan requirements and accumulate sufficient savings for housing affordability. These findings suggest that the current structure of the HPF may inadvertently reinforce existing socioeconomic inequalities rather than mitigate them.

This study makes two contributions to the existing literature on housing policies and subjective wellbeing. First, by shifting the focus from overall outcomes to equity considerations in evaluating the HPF, this research addresses a critical gap in understanding the distributional effects of housing policies. Second, this study contributes to the broader discourse on urban social stratification by illuminating how universal housing policies can inadvertently reinforce existing socioeconomic inequalities. By demonstrating that the HPF's benefits are not evenly distributed across urban populations, the research highlights the complex interplay between housing policies, migration status, and subjective wellbeing. This insight is particularly valuable in the context of China's rapid urbanization and the challenges faced by migrant workers in accessing urban amenities and social benefits.

These findings have several important policy implications. First, we find that HPF participation is associated with higher happiness, indicating that the program supports residents' wellbeing. To enhance this effect, policies should promote HPF enrollment and participation. Second, the benefits of HPF are uneven, with non-migrants experiencing gains while migrants show no significant improvement. To reduce this gap, the system should introduce a voluntary contribution mechanism and flexible participation options for migrants and non-traditional workers, enabling them to access HPF benefits despite unstable employment. Third, migrants face difficulties in utilizing HPF loans due to short job tenures and strict eligibility requirements. Targeted measures, such as lowering minimum contribution periods, adjusting base amounts for economically disadvantaged groups, and streamlining fund withdrawal and loan application procedures can improve access, reduce financial constraints, and support migrants' integration into urban housing markets.

We acknowledge several limitations in this study. First, the cross-sectional nature of the data from the China Household Finance Survey limits our ability to establish causal relationships and observe changes in subjective wellbeing over time. A longitudinal study design would offer a more comprehensive understanding of the long-term effects of HPF participation. Second, while the study focuses on the dichotomy between migrant and non-migrant populations, it may not fully capture the heterogeneity within these groups, such as differences based on education levels, occupation types, or duration of urban residence. Future research could benefit from a more nuanced categorization of population subgroups. Third, the study relies on self-reported measures of subjective wellbeing, which, while widely used in happiness research, may be subject to reporting biases and cultural interpretations. Incorporating objective measures of wellbeing or using mixed-methods approaches could provide a more robust assessment of the HPF's impact.

## Data Availability

The original contributions presented in the study are included in the article/supplementary material, further inquiries can be directed to the corresponding author.
